# Rhein Inhibits Microglia-Mediated Neuroinflammation and Neuronal Damage of Alzheimer’s Disease via Regulating the Glutamine–Aspartate–Arginine–NO Metabolic Pathway

**DOI:** 10.3390/ijms26136404

**Published:** 2025-07-03

**Authors:** Bingqing Chi, Zhengyi Zhang, Zhixin Zhang, Han Zhang, Mengjun Tian, Ying Wang, Xiaoyan Gao

**Affiliations:** School of Chinese Materia Medica, Beijing University of Chinese Medicine, Beijing 102488, China

**Keywords:** microglia, Alzheimer’s disease, neuron, neuroinflammation, metabolomics, metabolic flux, Rhein

## Abstract

Microglia-mediated neuroinflammation is a key driver of Alzheimer’s disease (AD). In AD, microglia are activated and trigger an increased secretion of pro-inflammatory factors. Rhein, an anthraquinone compound extracted from rhubarb, has been shown to reduce the secretion of pro-inflammatory cytokines including TNF-α and IL-1β in activated microglia. However, the mechanism of rhein on microglia-mediated neuroinflammation and neuronal damage in AD remains unclear. In this study, we found that rhein improved behavioral abnormalities in AD rats and reduced the levels of inflammatory factors such as IL-1β, iNOS, and NO in the brain of AD rats. In the LPS-induced microglial model, rhein significantly reduced the levels of inflammatory factors to improve neuroinflammation. Untargeted metabolomics showed that the reprogramming of glutamine metabolism occurred in M1 microglia. Targeted metabolomics and ^13^C, ^15^N isotope tracing experiments demonstrated that rhein regulated the metabolite levels in the glutamine–aspartate–arginine metabolic pathway. Meanwhile, the upregulated expression of proteins such as GLS1 and GOT1 within this pathway was reversed by rhein. Furthermore, we found that the glutamine–aspartate–arginine metabolic pathway regulates the production of nitric oxide (NO, a neuroinflammatory mediator). Rhein alleviates neuronal damage by inhibiting the glutamine–aspartate–arginine–NO metabolic pathway. In conclusion, our study shows that rhein may inhibits NO production by regulating the glutamine–aspartate–arginine metabolic pathway in activated microglia, thereby inhibiting the neuroinflammation and neuronal damage in AD.

## 1. Introduction

Alzheimer’s disease (AD) is a progressive neurodegenerative disease caused by damage to the neurons in the brain [1]. Neuroinflammation is an important process in the neurodegeneration of AD, involved in a vicious cycle of amyloid deposition, neuronal damage, tangle formation, and death [2]. Microglia, the innate immune cell in the brain, are the primary players in neuroinflammation. Under pathological conditions of sustained inflammatory stimulation, microglia become over-activated and then release inflammatory mediators such as interleukin-1β (IL-1β), TNF-α, NO, reactive oxygen (ROS), etc., resulting in neuronal damage and neurotoxicity [3]. Blocking the inflammatory response and neuronal damage caused by hyperactive microglia has the potential to treat AD [4].

Microglia have various immune phenotypes, such as pro-inflammatory (M1) phenotype and non-inflammatory alternative activation (M2) phenotype, associated with their functions [5]. Recent studies have shown that the phenotypic and functional changes of immune cells are supported by adenosine 5’-triphosphate (ATP) generation through metabolic pathways [6]. For example, in the M1 microglia, cellular metabolism is reprogrammed from oxidative phosphorylation (OXPHOS) to aerobic glycolysis for energy generation, and the aerobic glycolysis regulates NLRP3 inflammasome activation, which induces the IL-1β TNF-α production [7]. This metabolic switch eventually causes glycolytic pyruvate to be diverted to lactate instead of acetyl-CoA, further increasing glutamine consumption to supply the tricarboxylic acid (TCA) cycle [8]. In the state of M1 polarization, the TCA cycle is broken downstream of succinate, resulting in succinate accumulation [9]. Accumulated succinate directs hypoxia-inducible factor 1α (HIF1α)-mediated upregulation of glycolysis and IL-1β production [10]. In addition, arginine metabolism is shifted towards the inducible nitric oxide synthase (iNOS)-catalysed process, characterized by increased production of inflammatory mediator NO [11]. Some studies have indicated that the aspartate–arginosuccinate shunt connects the fragmented TCA cycle metabolites with NO production by arginine metabolism during the inflammatory response in the M1 macrophage [12]. Moreover, the biosynthesis of aspartate is mainly produced by the glutamate (the product of glutamine) and oxaloacetate in the TCA cycle under the catalysis of glutamic oxalacetic transaminase (GOT). The large number of inflammatory factors (such as NO, IL-1β) and reactive oxygen species (ROS) released by activated microglia can directly damage neurons, leading to neuronal dysfunction, synaptic loss, and even cell apoptosis [13]. As an important regulator in the nervous system, NO generate peroxy-nitrite (ONOO^−^) through oxidative stress and nitrative stress pathways [14]. ONOO^−^ can cause nitration modification of proteins, leading to their functional impairment [15]. This reaction also triggers lipid peroxidation, further exacerbating the metabolic imbalance of neurons, resulting in DNA structure damage and the apoptosis mechanism activated thereby participating in the pathological process of Alzheimer’s disease [16,17]. Therefore, we speculate that the glutamine–aspartate–arginine–NO metabolic pathway in activated microglia is associated with neuroinflammation and neuronal injury, and modulating this metabolic pathway in activated microglia may serve as a potential therapeutic strategy for AD.

Rhein, an anthraquinone compound extracted from traditional Chinese medicine (TCM) rhubarb, exhibits anti-neuroinflammatory activity by reducing the secretion of pro-inflammatory cytokines including TNF-α and IL-1β in LPS-stimulated activated microglia [18]. Rhein was detected in the cerebrospinal fluid (CSF) of patients with traumatic brain injury, indicating that rhein can pass through the damaged blood–brain barrier [19]. The potential of rhein has been also observed in many modern experimental models. In senescence-accelerated mouse prone-8 (SAMP8) mice, rhein lysinate (RHL) reduces the Aβ aggregation by reducing the levels of TNF-α and IL-6 and inhibiting oxidative stress in brain tissues [20]. In APP/PS1 mice, rhein significantly reduced Aβ burden and neuroinflammation, and eventually ameliorated cognitive impairment [21]. Our previous studies have shown that rhein exerts anti-neuroinflammatory activity by inhibiting the activation of the NLRP3 inflammasome in the brain of APP/PS1 mice. Rhein alleviates epilepsy and exerts neuroprotective effects by inhibiting the TLR4-NF κB signaling pathway [22]. As the microglia, whose functions are associated with metabolic reprogramming, play an important role in neuroinflammation of AD, it is important to explore the mechanism of rhein on microglia-mediated neuroinflammation and neuronal damage in AD. However, related research on the mechanism of rhein on microglia-mediated neuroinflammation and neuronal damage in AD remains limited.

Here, we aimed to explore the underlying mechanism of rhein on activated microglia-mediated neuroinflammation and neuronal damage in AD. Firstly, we investigated the anti-neuroinflammatory activity of rhein via in vitro and in vivo models. Secondly, we determined the metabolic pathway regulated by rhein in the activated microglia using untargeted metabolomics and metabolomics. Next, the metabolic pathway was verified by metabolic flux analysis and critical protein expression analysis. Then, the regulatory effect of the pathway on NO production was investigated using the pathway enzyme inhibitor. Finally, the protective effect of rhein on neurons was studied by culturing neurons in microglial conditioned medium. Our study shows that rhein may inhibit NO production by regulating the glutamine–aspartate–arginine metabolic pathway in activated microglia, thereby protecting neurons, reflecting the potential to treat activated microglia-mediated neuroinflammation and neuronal damage in AD.

## 2. Results

### 2.1. Effect of Rhein on Activated Microglia-Mediated Neuroinflammation in AD Rat Brain

**Figure 1:**
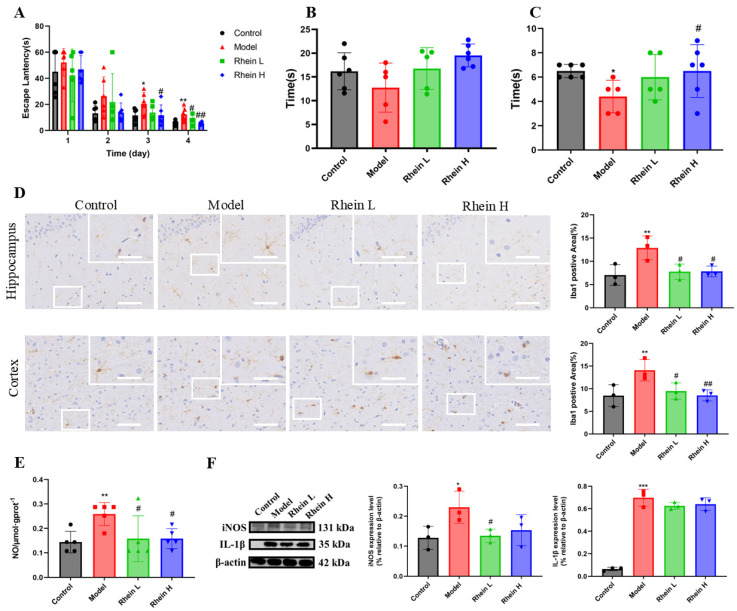
Rhein improved the learning and memory ability of AD rats by downregulating the expression of inflammatory mediators in the hippocampus of AD rats. (**A**) Escape latency in localized navigation test of rats after administration of rhein. Compared with the control group, ** *p* < 0.01, * *p* < 0.05. Compared with the model group, ^##^ *p* < 0.01, ^#^ *p* < 0.05. (**B**) Exercise time in target quadrant in spatial exploration experiment of rats after administration of rhein. (**C**) The times for crossing the platform area in the spatial exploration experiment of rats after administration of rhein. Compared with the control group, * *p* < 0.05. Compared with the model group, ^#^ *p* < 0.05. (**D**) Iba-1 immunohistochemical staining (left) and quantitative Iba1-positive areas in the hippocampal and cortex regions (right). Scale bar: 200 μm. The insets show the magnified microglia. Scale bar: 50 μm. Compared with the control group, ** *p* < 0.01. Compared with the model group, ^##^ *p* < 0.01, ^#^ *p* < 0.05. (**E**) The level of NO in the hippocampus of each group. Compared with the control group, ** *p* < 0.01. Compared with the model group, ^#^ *p* < 0.05. (**F**) The expression of iNOS and IL-1β in the hippocampus of rats in each group was determined by Western blot. Compared with the control group, *** *p* < 0.001, * *p* < 0.05. Compared with the model group, ^#^ *p* < 0.05.

Accumulating evidence demonstrates that the neuroinflammation contributes to cognitive decline associated with AD [23]. To investigate the anti-inflammatory effect of rhein, we first explored whether rhein treatment saved cognitive impairment in AD rats. The Morris water maze (MWM) test was performed to evaluate learning and memory ability of AD rats. The MWM test revealed that the escape latency was progressively decreased in all tested groups during the 4 days acquisition period. AD rats untreated showed an elevated escape latency during the 4 days acquisition compared to control rats (Figure 1A). In contrast, AD rats treated with rhein demonstrated significantly diminished escape latency on days 3 and 4 compared to untreated AD rats (Figure 1A). In addition, to provide evidence for spatial learning, after the final training session, a reference memory test (spatial probe test) was performed and the rats were tested without a platform to escape. The results showed that the rats in the high dose group spent more time in the target quadrant than the AD rats (Figure 1B). Platform crossing times were significantly higher in the high-dose group than in the AD rats (*p* < 0.05) (Figure 1C).

The activation of microglia is the main feature of neuroinflammation [2]. To further investigate the effect of rhein on neuroinflammation, we detected the activation situation of microglia in the brains of AD rats. The total number of microglial cells, as an activation hallmark, was analyzed using Iba-1 staining. The results showed that, in the model group, the area of Iba-1 positive regions in the hippocampus and cortex increased significantly (*p* < 0.01), and the rhein treatment significantly reduced the area of Iba-1 positive regions (Figure 1D, Rhein L, 20 mg/kg; Rhein H, 40 mg/kg). Our findings suggest that rhein can inhibit the activation of microglia in the brain of AD rats.

Next, in order to evaluate the effect of rhein on microglia-mediated neuroinflammation in vivo, the NO, iNOS, and IL-1β in the hippocampus of AD rats were detected. The results showed that the rhein significantly reduced NO generation (*p* < 0.05) (Figure 1E), and reduced the expression of IL-1β and iNOS in rat hippocampus (Figure 1F), indicating that rhein has an anti-neuroinflammatory activity in vivo.

These results showed that the rhein inhabited the neuroinflammation in AD rat brain, thus improving the cognitive deficits in AD rats.

### 2.2. Rhein Inhibits LPS-Induced Inflammatory Response Mediated by Activated Microglia

**Figure 2:**
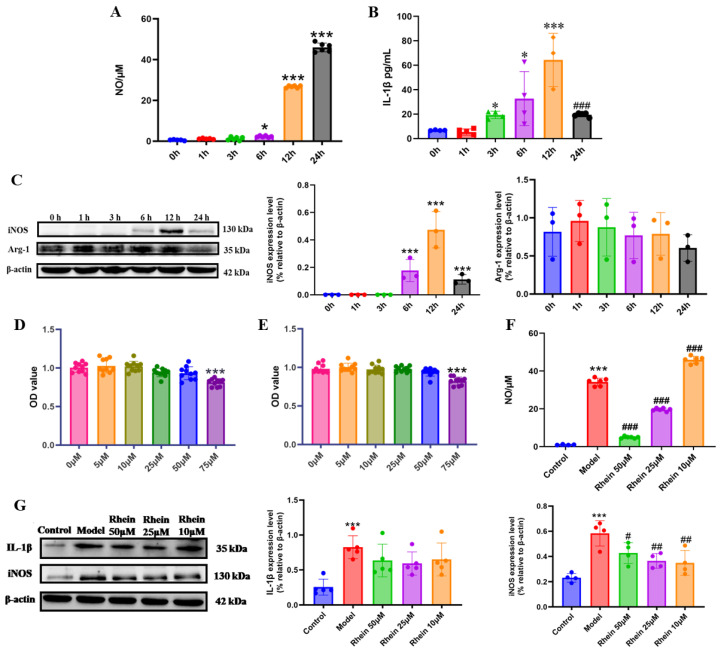
Rhein inhibited the neuroinflammatory response by downregulating the expression of inflammatory mediators in LPS-stimulated BV2 cells. Levels of NO (**A**) and IL-1β (**B**) at different time points stimulated by LPS. Compared with the 0 h Group, *** *p* < 0.001, * *p* < 0.05. Compared with the 12 h group, ^###^ *p* < 0.001. (**C**) Expression of iNOS and Arg-1 at different time points under LPS stimulation was determined by Western blot. Compared with the 0 h Group, *** *p* < 0.001. The cell viability assessed using MTT pretreated with or without different concentrations of rhein (0, 5, 10, 25, 50, and 75 μM) for 6 h (**D**) and 12 h (**E**) in BV2 cell. Compared with the 0 μM Group, *** *p* < 0.001. (**F**) Effect of rhein on NO secretion of BV2 cells. Compared with the control group, *** *p* < 0.001. Compared with the model group, ^###^ *p* < 0.001. (**G**) Western blotting analysis of the level of inflammatory mediators IL-1β, and iNOS in BV2 cells. Compared with the control group, *** *p* < 0.001. Compared with the model group, ^##^ *p* < 0.01, ^#^ *p* < 0.05.

Activated microglia are the main source of proinflammatory cytokines that damage neurons in the brain [24]. In order to facilitate the exploration of the mechanisms underlying the role of rhein in microglia-mediated neuroinflammation, we conducted research at the cellular level. We first established an inflammation model of M1 activated microglia. BV2 cells were stimulated with LPS at different time points, and the markers of M1 activation, including IL-1β, iNOS, and NO, and the marker of M2 activation, Arg-1, were detected [18]. The results showed that the NO increased significantly with time (Figure 2A). The expression levels of IL-1β (Figure 2B) and iNOS (Figure 2C) were the highest at 12 h (*p* < 0.001). The expression level of Arg-1 was not significantly different within 24 h (Figure 2C). According to the expression levels of M1 and M2 activation markers, LPS stimulation for 12 h was investigated to establish the model of M1 microglia.

Then, we evaluated the safe dose of rhein (5, 10, 25, 50, and 75 μM) in BV2 cells and primary microglia. The MTT results showed that rhein at a concentration of ≤50 μM induced no toxicity in BV2 cells and primary microglia (Figure 2D,E and Supplementary Figure S1A,B). 50 μM, 25 μM, and 10 μM rhein were selected for the following study. We further examined the effects of rhein on the levels of NO, iNOS, and IL-1β. The results showed that rhein treatment significantly inhibited LPS-induced NO production in both BV2 cell and primary microglia (Figure 2F, Supplementary Figure S1C), as well as the levels of iNOS in BV2 cell (Figure 2G). These data showed that rhein effectively inhibited the inflammatory response mediated by microglia.

### 2.3. Regulatory Effect of Rhein on Metabolism of Activated Microglia

As the microglial functions are related to the metabolic reprogramming, we first investigate the metabolic changes of M1 microglia by untargeted metabolomics to discover the metabolic pathways which are related to neuroinflammation. Then we confirmed the metabolic pathway which rhein regulated by targeted metabolomics.

**Figure 3:**
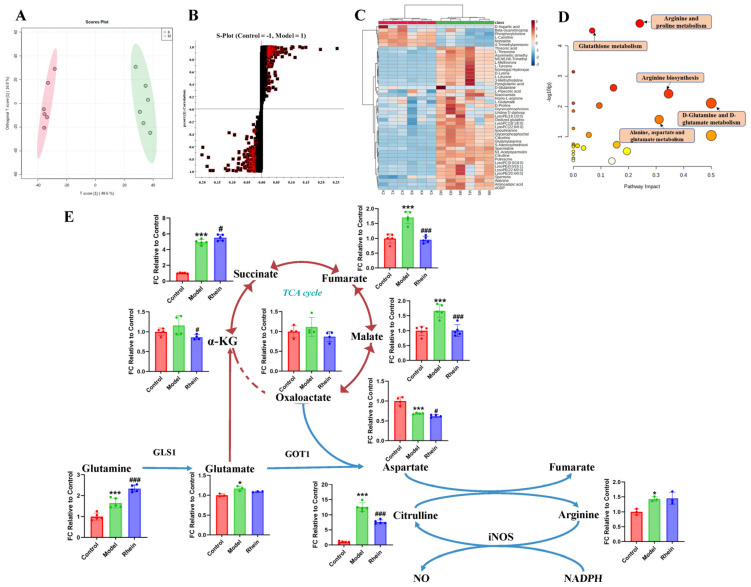
Regulatory effect of rhein on metabolism of activated M1 microglia. (**A**) OPLS-DA of primary microglia between control group and LPS group (R^2^ = 0.993, Q^2^ = 0.961). (**B**) S-Plot of primary microglia between control group and LPS group (VIP > 1, *p* < 0.05). The square pattern with a red border represents VIP > 1 and *p* < 0.05. (**C**) Differential metabolites of primary microglia between control group and LPS group. (**D**) Differential metabolite pathway of primary microglia between control group and LPS group. Node colors are based on P-values, with red ranging from light to dark and P-values ranging from large to small. The size of a node is based on its path influence value, from small to large, and from small to large in influence value. (**E**) The levels of metabolites of the glutamine–aspartate–arginine metabolic pathway in primary microglia. Compared with the control group, *** *p* < 0.001, * *p* < 0.05, compared with the model group, ^###^ *p* < 0.001, ^#^ *p* < 0.05.

To investigate the metabolic changes in M1 microglia, we analyzed the intracellular microglia metabolites by untargeted metabolomics in cultured primary microglia with LPS treatment. We found that the metabolic profiles between the control group and LPS group were completely different (Figure 3A and Supplementary Figure S2), indicating that the metabolic reprogramming occurred after LPS treatment. A total of 46 differential metabolites between the two groups were screened and identified (Figure 3B,C, Supplementary Table S1). Metabolite set enrichment analysis of differentially abundant metabolites revealed that the glutamine and glutamate metabolic pathway was one of the disturbed metabolic pathways, indicating that this pathway may be closely related to the inflammatory response of microglia (Figure 3D). Downstream regulation of the glutamine and glutamate metabolism includes alanine, aspartate, and glutamate metabolism, arginine and proline metabolism, and arginine biosynthesis. These pathways were all disturbed to varying degrees, suggesting that glutamine metabolic reprogramming exists in M1 microglia.

Based on the results for untargeted metabolites, it has been shown that glutamine metabolic reprogramming occurred in M1 microglia. Combined with literature research, we speculated that the glutamine–aspartate–arginine metabolic pathway is related to microglia-mediated neuroinflammation. To confirm the metabolic pathway regulated by rhein in M1 microglia, targeted metabolomics was used to analyze the levels of metabolites in the glutamine–aspartate–arginine metabolic pathway. We found that the concentration of glutamine, glutamate, arginine and citrulline were significantly increased by LPS treatment. Furthermore, the intermediate metabolites of the TCA cycle, such as succinate, fumarate, and malate, were significantly increased during LPS treatment in microglia (Figure 3E). In addition, we discovered that the level of aspartate decreased significantly, speculating that aspartate consumption rate may be faster than its production. Thus, the metabolite abundance in the glutamine–aspartate–arginine metabolic pathway was upregulated in LPS treatment. After rhein treatment, the levels of the intermediate metabolites of TCA cycle, such as α-KG, fumarate, and malate, were significantly decreased, indicating that rhein reversed the levels of these intermediate metabolites (Figure 3E). In addition, we found that the level of aspartate and citrulline were significantly decreased by rhein treatment, speculating that aspartate biosynthesis and the metabolism from arginine to citrulline were down-regulated.

These results indicate that the glutamine–aspartate–arginine metabolic pathway may be related to neuroinflammation, and rhein reversed the levels of metabolites in this metabolic pathway.

### 2.4. Regulatory Effect of Rhein on the Glutamine–Aspartate–Arginine Metabolic Pathway in Activated Microglia

**Figure 4:**
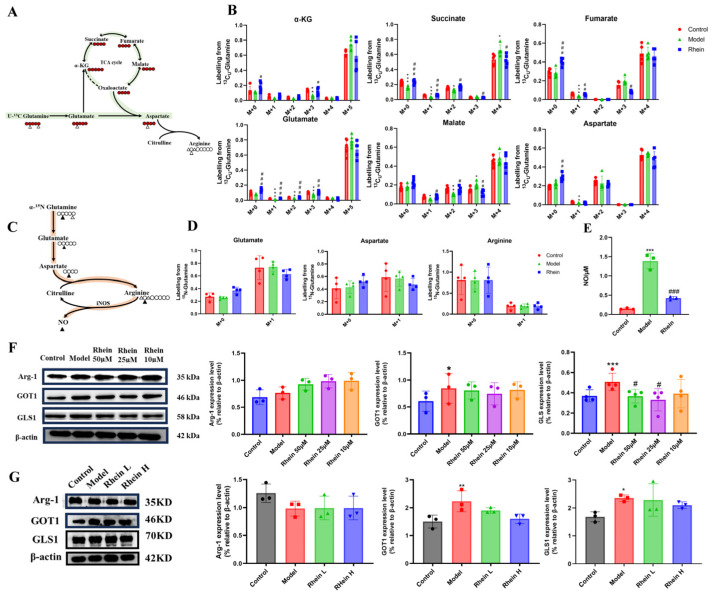
The changes in metabolic flux and the expression of enzymes in the glutamine–aspartate–arginine metabolic pathway. (**A**) Schematic model of glutamine–aspartate–arginine metabolic pathway. Red circles represent carbons derived from [U-^13^C_5_]-glutamine, and black circles are unlabeled. (**B**) Mass isotopologue distributions of AKG, Succinate, Fumarate, Malate, Citric acid, Glutamate, and Aspartate. Compared with the control group, *** *p* < 0.001, ** *p* < 0.01, * *p* < 0.05. Compared with the model group, ^###^ *p* < 0.001, ^##^ *p* < 0.01, ^#^ *p* < 0.05. (**C**) Schematic model of glutamine–aspartate–arginine metabolic pathway. Black triangles represent nitrogens derived from ^15^N-glutamine. (**D**) Mass isotopologue distributions of glutamate, aspartate, and arginine. (**E**) The level of NO in different groups. Compared with the control group, *** *p* < 0.001. Compared with the model group, ^###^ *p* < 0.001. (**F**) Arg-1, GOT1, and GLS1 protein expression in BV2 cells. Compared with the control group, *** *p* < 0.001, * *p* < 0.05. Compared with the model group, ^#^ *p* < 0.05. (**G**) Arg-1, and GOT1, GLS1 protein expression in brain tissue. Compared with the control group, ** *p* < 0.01, * *p* < 0.05. Compared with the model group, ^#^ *p* < 0.05.

To verify the effect of rhein on the glutamine–aspartate–arginine metabolic pathway, we cultured BV2 cells with [U-^13^C_5_]-glutamine and ^15^N-glutamine and traced the isotope-labeled intermediates in this metabolic pathway. M + 0 represents metabolites that have not been labeled with isotopes. M + 1, M + 2, M + 3, M + 4, and M + 5, respectively, indicate that 1, 2, 3, 4, and 5 carbon atoms in the metabolites are labeled with ^13^C. We found that LPS treatment promoted metabolic flux of [U-^13^C_5_]-glutamine to the intermediate metabolites of TCA cycle, such as α-KG M + 5, malate M + 4, and succinate M + 4, and also for glutamate M + 5 and aspartate M + 4 (Figure 4A,B). It is suggested that direct glutamine contribution to the downstream carbon flux in the TCA cycle and aspartate biosynthesis were increased. After rhein treatment, we discovered that rhein attenuated the enhanced glutamine derived from the intermediate metabolites of the TCA cycle, such as α-KG M + 5, malate M + 4, and succinate M + 4. In addition to the TCA cycle intermediates, we observed a reduced contribution of glutamine to aspartate, which is derived predominantly from glutamine via the TCA cycle (Figure 4A,B). To monitor nitrogen transfer in the glutamine–aspartate–arginine metabolic pathway, we conducted a metabolic flux assay using ^15^N (amino-nitrogen)-labeled glutamine, which transfers ^15^N to glutamate, aspartate, arginine, and NO merely though enzymatic reaction [25]. We found that LPS treatment promoted metabolic flux of ^15^N-glutamine to glutamate M + 1 and arginine M + 1 (no significance), while the NO production was increased significantly (Figure 4C–E). The level of aspartate M + 1 was reduced as its consumption was higher than its production. After rhein administration, we discovered that rhein attenuated the enhanced glutamine-derived glutamate M + 1 and arginine M + 1 (no significance), while the NO production was significantly decreased (Figure 4C–E). We also observed a reduced contribution of glutamine to aspartate, which suggested that aspartate biosynthesis was decreased by rhein treatment. These data suggest that rhein reversed the upregulated glutamine–aspartate–arginine metabolic pathway, which is consistent with the metabolomics results.

To further verify the effect of rhein on the glutamine–aspartate–arginine metabolic pathway, we studied the enzymes involved in this pathway in BV2 cells. We found that the expression of GLS1 and GOT1 was significantly increased in LPS group (*p* < 0.05) (Figure 4F). The data supported that the increased expression of GLS1 and GOT1 might result in the accumulation of the intermediate metabolites of TCA cycle and the sufficiency of aspartate. Moreover, the expression of GLS1 and GOT1 was decreased by rhein treatment, with reduced glutamate-to-TCA cycle and aspartate biosynthesis conversion (Figure 4F), supporting the metabolomics and metabolic flux results in Figure 3E and Figure 4A–E. In addition, we also measured the expression of GLS1, GOT1 and Arg-1 in the brain of AD rats. The results showed that rhein also reversed the levels of GLS1 and GOT1 in the AD rat brain (Figure 4G).

These data supported that rhein regulated the glutamine–aspartate–arginine metabolic pathway through down-regulating its protein GLS1 and GOT1 expression.

### 2.5. The Glutamine–Aspartate–Arginine–NO Metabolic Pathway Regulates the Inflammatory Factor NO Production in Microglia

In the above experiments, we demonstrated that the expression of proteins such as GLS1 in M1 microglia regulates the glutamine aspartate arginine metabolic pathway. At the same time, we observed a shift in arginine metabolism towards NO production. NO is an important inflammatory factor in AD neuroinflammation, so we investigated whether the metabolic pathway of glutamine aspartate arginine regulates the production of inflammatory mediator NO.

**Figure 5:**
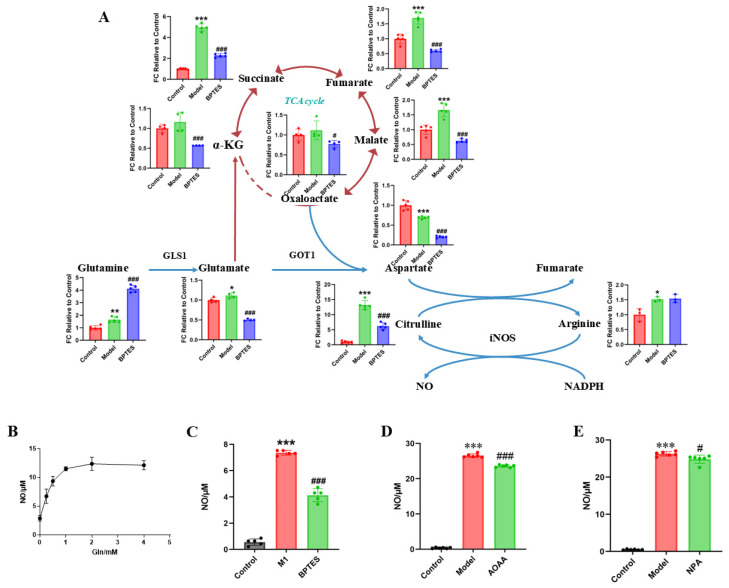
Glutamine metabolism regulates downstream aspartate biosynthesis and regulates the production of inflammatory mediator NO. (**A**) The levels of metabolites of the glutamine–aspartate–arginine metabolic pathway in primary microglia were relative to the FC value of the control group. Compared with the control group, *** *p* < 0.001, ** *p* < 0.01, * *p* < 0.05. Compared with the model group, ^###^ *p* < 0.001, ^#^ *p* < 0.05. (**B**) The production of NO in different glutamine concentrations. The effects of BPTES (**C**), AOAA (**D**) and NPA (**E**) on NO production in BV2 cells. Compared with control group, *** *p* < 0.001. Compared with the model group, ^###^ *p* < 0.001, ^#^ *p* < 0.05.

In this pathway, glutamine is the main carbon and nitrogen source of the TCA cycle and downstream metabolites [26]. First, to verify the contribution of glutamine to the downstream metabolites, we used targeted metabolomics to analyze the effect of BPTES (GLS1 enzyme inhibitors) on the levels of metabolites in the pathway (Figure 5A). We found that the concentration of glutamine was significantly increased by BPTES treatment. Meanwhile, BPTES treatment significantly reduced the levels of intermediate metabolites of the TCA cycle, suggesting that glutamine is an important substrate of the TCA cycle in activated microglia. In addition, the levels of aspartate and citrulline decreased significantly, and no significant differences in the level of arginine were observed, which suggested that the metabolism of arginine to citrulline was inhibited due to the reduction in the amount of aspartate. These results suggest that glutamine is the key substrate of metabolites in the TCA cycle and aspartate biosynthesis.

As the arginine metabolism, the downstream glutamine–aspartate–arginine metabolic pathway, produces inflammatory mediator NO, we investigated the effect of glutamine on NO production. The BV2 cells were cultured in medium containing different concentrations of glutamine. We found that the level of NO produced by LPS-stimulated BV2 cells was glutamine dose-dependent to some extent (Figure 5B). To further explore the effect of the glutamine–aspartate–arginine metabolic pathway on NO production in BV2 cells, we used the GLS1 enzyme inhibitor BPTES, the succinate dehydrogenase inhibitor 3-nitropropionic acid (NPA), and GOT1 inhibitor aminooxyacetic acid hemihydrochloride (AOAA) to inhibit the enzyme in this pathway. We found that the level of NO produced by BV2 cells increased significantly under LPS stimulation (*p* < 0.001), while NO decreased significantly after pretreatment with enzyme inhibitor (Figure 5C–E). It is worth noting that the decrease in NO production levels after BPTES treatment is more significant than that after AOAA and NPA treatment, which may be related to the rate limiting position of GLS1 in the glutamine pathway. These results suggest that glutamine–aspartate–arginine metabolic pathway plays an important role in the regulation of NO production.

In conclusion, it is suggested that activated microglia regulate the inflammatory factor NO production through the glutamine–aspartate–arginine–NO metabolic pathway, leading to neuroinflammation.

### 2.6. Rhein Alleviates Neuronal Damage by Inhibiting the Glutamine–Aspartate–Arginine–NO Metabolic Pathway

As mentioned above, we have demonstrated that rhein down-regulates the glutamine–aspartate–arginine–NO metabolic pathway and inhibits NO production in microglia, thereby suppressing microglia-mediated neuroinflammation. NO produced by this pathway regulation is an important inflammatory mediator that can directly damage neurons or exacerbate neuronal damage by producing ONOO^−^, leading to neuronal dysfunction and even cell apoptosis [27,28]. Therefore, we then investigated the effect of rhein on neuronal damage mediated by activated microglia.

**Figure 6:**
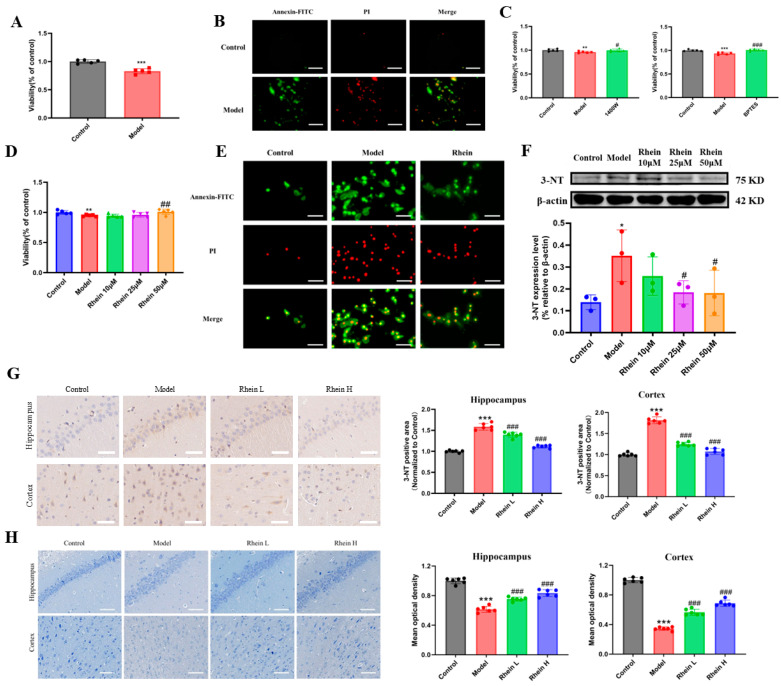
The protective effect of rhein on conditioned medium-induced primary neurons and in brain of AD rats. (**A**) Viability of PC12 cells cultured in BV2 microglia conditioned medium. Compared with control group, *** *p* < 0.001. (**B**) Apoptosis of PC12 cells cultured in BV2 microglia conditioned medium (Scale 50 μm). (**C**) Viability of PC12 cells cultured in inhibitors 1400 W and BPTES mediated BV2 microglia conditioned medium. Compared with control group, *** *p* < 0.001, ** *p* < 0.01. Compared with the model group, ^###^ *p* < 0.001, ^#^ *p* < 0.05. (**D**) Viability of PC12 cells cultured in BV2 microglia conditioned medium by intervention of rhein. Compared with control group, ** *p* < 0.01. Compared with the model group, ^##^ *p* < 0.01. (**E**) Apoptosis of PC12 cells cultured in BV2 microglia conditioned medium by intervention of rhein (Scale 50 μm). (**F**) Level of 3-NT in PC12 cells cultured in BV2 microglia conditioned medium intervention of rhein. Compared with control group, * *p* < 0.05. Compared with the model group, ^#^ *p* < 0.05. (**G**) Level of 3-NT in brain tissue by AD rats (Scale 50 μm). Compared with the control group, *** *p* < 0.001. Compared with the model group, ^###^ *p* < 0.001. (**H**) Nissl staining of brain tissue by AD rats (Scale 50 μm). Compared with the control group, *** *p* < 0.001. Compared with the model group, ^###^ *p* < 0.001.

In order to determine the interaction between BV2 microglia and PC12 cells, PC12 cells were cultured in BV2 microglia conditioned medium, and the cell activity and apoptosis of PC12 cells was determined. The results indicated that, when PC12 cells were cultured in LPS-induced BV2 microglia conditioned medium, the activity of PC12 cells in the BV2 microglia conditioned medium group was significantly decreased (*p* < 0.001) (Figure 6A). In addition, the results of apoptosis showed that the amount of green and red fluorescence in PC12 cells in BV2 microglia conditioned medium group treated with LPS increased, indicating that the cells were undergoing early and late apoptosis, respectively (Figure 6B). As an iNOS inhibitor, 1400 W can directly inhibit the production of NO mediated by iNOS enzyme by inhibiting the activity of iNOS enzyme. GLS1 enzyme inhibitor BPTES can inhibit the level of NO production by inhibiting the glutamine–aspartate–arginine metabolic pathway. The results showed that the cell activity of PC12 cells cultured in conditioned medium increased significantly (*p* < 0.05, or *p* < 0.001) after BV2 microglia were pretreated with 1400 W or BPTES (Figure 6C). The above results indicate that LPS-induced BV2 microglia produce inflammatory factor NO by regulating the glutamine–aspartate–arginine metabolic pathway, reducing the activity of PC12 cells and promoting apoptosis of PC12 cells.

Subsequently, to explore the protective effect of rhein on BV2 microglia-mediated neuronal damage, we measured the effect of rhein on the activity and apoptosis of PC12 cells. The activity of PC12 cells in the LPS-treated medium group decreased significantly (*p* < 0.05), while that in the 50 μM rhein-pretreated medium group increased significantly (*p* < 0.01) (Figure 6D). The results of fluorescence imaging showed that the apoptosis of PC12 cells in the BV2 microglia conditioned medium group was increased by LPS, and the apoptosis level in PC12 cells in the BV2 microglia conditioned medium group was decreased by 50 μM rhein (Figure 6E). NO produced by BV2 microglia can produce 3-NT through a series of reactions, and the level of 3-NT can reflect the level of nitration stress in the brain, so we used Western Blot to determine the level of 3-NT in different groups of cells. The results showed that the level of 3-NT in PC12 cells cultured in conditioned medium of BV2 microglia treated with LPS increased significantly (*p* < 0.05). The level of 3-NT in PC12 cells pretreated with rhein of 25 μM and 50 μM was significantly decreased (*p* < 0.05) (Figure 6F). A similar phenomenon was observed in the brain of AD rats, and the 3-NT positive region decreased after rhein administration (Figure 6G). Nissl staining results showed that rhein intervention increased the number of neurons in the hippocampus compared with model rats, and the cells were closely arranged and normal in shape (Figure 6H).

The results showed that rhein ameliorated the decrease in neuronal viability and apoptosis, and reduceed the level of nitration stress by inhibiting the glutamine–aspartate–arginine–NO metabolic pathway to protect PC12 cells and neurons in the brain of AD rats.

## 3. Discussion

**Figure 7:**
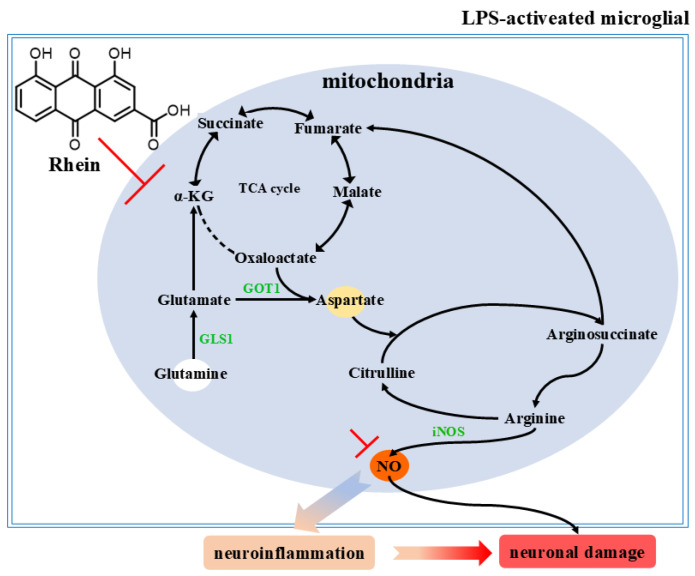
Rhein inhibits the glutamine aspartate arginine NO metabolic pathway and pathway proteins, thereby alleviating neuroinflammation and neuronal damage.

In this study, an LPS-induced M1 microglia inflammation model and an AD rat model were used to explore the potential mechanism of rhein on microglia-mediated neuroinflammation and neuronal damage. We have demonstrated that rhein has anti-neuroinflammatory activity in M1 microglial cells and AD rat model. Moreover, we found that rhein exerts an anti-inflammatory effect on AD neuroinflammation by reversing the glutamine–aspartate–arginine–NO metabolic pathway and thus inhibiting NO production. More importantly, we have demonstrated that rhein improves neuronal activity reduction, apoptosis, and neuronal nitrification stress levels by inhibiting neuroinflammation induced by microglia, thereby alleviating neuronal damage (Figure 7).

Neuroinflammation mediated by overactivated microglia plays an important role in the pathogenesis of AD [29]. Inflammatory (M1) microglia release inflammatory mediators, including IL-1β, iNOS, and NO [30,31]. In the CNS, IL-1β overexposure damage neurons, blood vessels, and oligodendrocytes. iNOS, as a pro-inflammatory marker, is induced to express in M1 microglia, and overexpression of iNOS leads to the NO generation. The reaction of NO with the superoxide anion produces peroxy-nitrite, which is an inflammatory mediator that damage neurons. Our study shows that rhein inhibited the levels of inflammatory mediators IL-1β and iNOS and inhibited the production of NO in M1 microglia and the AD rat model, indicating that rhein has anti-neuroinflammatory activity.

Metabolic reprogramming occurs in microglia in the LPS stimulation. In our study, the glutamine–aspartate–arginine metabolic pathway was related to inflammatory response of activated microglia. We demonstrated that rhein has a regulatory effect on this pathway. Glutamine is an important amino acid that supplies carbon and nitrogen to fuel biosynthesis [32]. Our results showed that LPS promoted the formation of α-KG from glutamate, which further promoted glutamine-derived metabolites from entering the TCA cycle, consequently increasing precursors for aspartate biosynthesis. This process is related to the phenotypes and functions of microglia, and is essential to the pathogenesis of AD [26]. After rhein treatment, the ability of glutamine to replenish the TCA cycle was reduced, and the levels of intermediate metabolites in TCA cycle were reversed. In addition, it has been reported that aspartate production is dependent on the amidogen from glutamine and the carbon skeleton from oxaloacetate in the TCA cycle [33]. Aspartate can partake in arginine regeneration in conjunction with citrulline for sustained NO production by iNOS of M1 microglia [34]. These are consistent with our results that LPS promoted the production and consumption of aspartate, and then accelerated the production of arginine and NO, supporting that aspartate biosynthesis is an important node in the glutamine–aspartate–arginine metabolic pathway. After rhein treatment, we found that aspartate biosynthesis was decreased, and the production of arginine and NO were also reduced. Therefore, our results suggest that rhein represents a potential therapeutic value for AD neuroinflammation by adjusting the glutamine–aspartate–arginine metabolic pathway.

It is reported that nitrogen from glutamine was transferred to aspartate, and nitrogen from aspartate was distributed into arginine to support NO production [33]. NO, as an inflammatory mediator, damages neurons through a variety of pathways. For example, NO and superoxide produces peroxynitrite by diffusion-controlled reaction [35]. Peroxynitrite can cause nitrotyrosylation of proteins, leading to their dysfunction. It interacts with lipids and DNA through direct oxidation reaction or indirect free mechanism, causing cell damage [36]. Therefore, we further investigated the relationship between the glutamine–aspartate–arginine metabolic pathway and NO. In this study, we used enzyme inhibitors of this pathway to test whether the glutamine–aspartate–arginine metabolic pathway regulates the production of NO. BPTES is a well-known glutaminase inhibitor that effectively blocks the utilization of glutamine. Our study revealed that BPTES treatment significantly diminished LPS-induced NO production in microglia, thereby highlighting the crucial regulatory role of glutamine metabolism in NO production. NPA can inhibit succinate dehydrogenase in the TCA cycle [37], thereby restraining the production of aspartate from glutamine indirectly. Similarly, AOAA can inhibit GOT1, a key enzyme functioning in the aspartate–arginosuccinate shunt [12], thereby restraining aspartate biosynthesis. Both NPA and AOAA treatments effectively reduced NO production in activated microglia, underscoring the significant regulatory role of aspartate biosynthesis in NO production. Collectively, these findings suggest that the glutamine–aspartate–arginine–NO metabolic pathway plays a pivotal role in regulating NO production. Therefore, modulating this metabolic pathway holds great promise for inhibiting NO production in activated microglia.

Neuroinflammation can lead to neuronal damage, and NO plays an important role in this process. NO induces neuronal apoptosis by activating cGMP-dependent protein kinase (PKG) or NF-κB signaling pathways [38]. Furthermore, NO can also induce tyrosine nitration of key proteins such as α-tubulin, thereby disrupting the homeostasis of cytoskeletal proteins and ultimately leading to apoptosis [39]. Our findings demonstrated a significant decrease in neuronal activity and a significant increase in apoptosis in neurons cultured with conditioned medium from activated microglia. The neuronal injury and apoptosis mediated by microglial neuroinflammation were reversed by rhein intervention. 3-NT is a product of protein tyrosine residues nitration and is commonly used as a biomarker of oxidative stress and nitration stress [40]. NO reacts with superoxide anion (O_2_^−^) in vivo to form ONOO^−^, which can react with tyrosine residues in proteins to form 3-NT [14]. The accumulation of 3-NT can induce apoptosis or necrosis and further aggravate neuronal damage [41]. In our research, rhein was able to reduce the expression of 3-NT in PC12 cells and the brain of AD rats, thereby alleviating neuronal damage.

Our study revealed the metabolic changes of microglia induced by LPS, and further screened out the glutamine–aspartate–arginine–NO metabolic pathway related to neuroinflammation and neuronal damage in AD. Modulation of the microglial glutamine–aspartate–arginine–NO metabolic pathway may represent a promising therapeutic strategy for AD.

## 4. Materials and Methods

### 4.1. Chemicals and Reagents

Rhein (MW 284.22 of purity >98%, CAS: 478-43-3) was purchased from Beijing Tetrahedral Biotechnology Co., Ltd. (Beijing, China). Aβ_1–42_ (CAS: 107761-42-2 for lyophilized Aβ_1–42_) was obtained from Nanjing Peptide Biotech Co., Ltd. (Nanjing, China). α-KG (MW 146.10 of purity >98%, CAS: 328-50-7), Succinate (MW 118.09 of purity >98%, CAS: 110-15-6), Fumarate (MW 116.07 of purity >98%, CAS: 110-17-8), Malate (MW 134.09 of purity >98%, CAS: 97-67-6), Glutamate (MW 147.13 of purity >98%, CAS: 56-86-0), Aspartate (MW 133.10 of purity >98%, CAS: 617-45-8), Arginine (MW 174.20 of purity >98%, CAS: 74-79-3) standards, and ^15^N-Glutamine (MW 161.16 of purity =98%, CAS: 80143-57-3) were purchased from Shanghai Yuanye Biotechnology Co., Ltd. (Shanghai, China). [U-^13^C_5_]-Glutamine (MW 151.11 of purity =98%, CAS: 184161-19-1) and LPS (Escherichia coli 0111: B4, CAS: 297-473-0) were obtained from Sigma (St. Louis, MO, USA). LC-MS grade acetonitrile, methanol, isopropanol, and formic acid were purchased from Thermo Fisher Scientific (Waltham, MA, USA). Dulbecco minimum essential medium (DMEM, CAT: C11995500BT), fetal bovine serum (FBS, CAT: 10099141C), 0.25% trypsin (CAT: 25200056) and penicillin–streptomycin (P/S, CAT: 15140122) were purchased from Gibco (Grand Island, NY, USA). The NO kit (CAT: A013-1-1) was purchased from Nanjing Jiancheng Bioengineering Institute (Nanjing, China). The IL-1β mouse Enzyme-linked immunosorbent kit (CAT: L201202315) was purchased from Wuhan Cloud-Clone Corp. (Wuhan, China).

### 4.2. Animal

Sprague Dawley (SD) rats were purchased from SPF (Beijing) Biotechnology Co., Ltd. (Beijing, China). The 1-day-old SD rats were used to obtain primary microglia, and the 2-month-old SD rats were used for the establishment of the AD rat model. The approval number is SCXK (Beijing) 2019-0010, and the rats were raised in the Animal Experimental Center of Beijing University of Chinese Medicine. The temperature was controlled at 21–25 °C. The relative humidity was 55–65%, and the cycle of day and night was 12 h alternately. The rats adapted to the environment for 7 days, during which they drank and ate freely.

To investigate the anti-neuroinflammatory activity of rhein on AD rats, using a random number table, SD rats were randomly divided into blank group, model group, low-dosed group, and high-dose group, with 10 rats in each group. We established an AD rat model by injecting Aβ_1–42_ into the rat hippocampus using the brain stereotaxic apparatus. SD rats were sedated with 1.5% sodium pentobarbital (2 mL/kg) before being placed on stereotaxic apparatus and having a midline sagittal incision made to expose the skull. Bregma and lambda were found and, using bregma as the midpoint point, two holes in the skull were produced using a drill machine at the stereotaxic co-ordinates of −4 mm antero-posterior, ±2 mm from the central line, and  −4 mm dorso-ventral from below the surface of the brain [42]. Aβ_1–42_ (2 μg/μL) was injected bilaterally into the CA1 region of the hippocampus using a 10 μL syringe (5 μL/side). Microinjections were performed for 5 min, and needles were left in place to facilitate the diffusion of the injected materials [43]. After the acclimation of the model, rhein (5 mg/mL) was injected intraperitoneally. The low-dosed group (Rhein L group) was intraperitoneally injected with 20 mg/kg, while the high-dosed group (Rhein H group) injected with 40 mg/kg [44]. The blank group rats were given normal saline in the same way.

### 4.3. Morris Water Maze

The Morris water maze test was used to evaluate the learning and memory ability of rats. All rats were trained and tested in a circular pool with a diameter of 121 cm, as described earlier [45]. During the 4 days of training, each rat was placed into the pool from different positions around the border of the pool and swam for 60 s. If the rat failed to find the platform within 60 s, they were guided to the platform to stay for 15 s, and the escape latency was recorded as 60 s. At 24 h after the last training trial, the platform was removed, and the rats were tested for memory retention in a probe trial. The number of times that the rats crossed the area of the platform and the time spent in the target quadrant were recorded. Throughout the experiment, each mouse’s swim path was tracked by a video tracking system (Noldus Information Technology Co., Ltd., Wageningen, The Netherlands), and its escape latency, time in the target quadrant, and target crossing times were also recorded.

### 4.4. Immunohistochemistry

To determine the activation of microglia and nitrification stress in the brain of AD rats in each group, ionized calcium-binding adaptor molecule 1 (Iba-1) and 3-nitrotyrosine (3-NT) immunohistochemistry was performed. The brain slices were incubated with anti-Iba-1 and anti-3-NT, and the secondary antibodies sequentially. In short, the brains of rats were embedded in paraffin, sectioned, and routinely dewaxed until anhydrous. Antigen repair on tissue slices was performed using the indirect high-pressure thermal repair method. Block endogenous peroxidase in tissue slices (5%BSA) and block at room temperature for 30 min. Add Iba-1 antibody (1:100) (or add 3-NT antibody 1:100) and incubate overnight at 4 °C. After washing with PBS, add secondary antibody (1:200) dropwise. After washing with PBS, DAB color was developed and counterstained with hematoxylin. Using ImageJ (version 1.54, NIH, Bethesda, MD, USA) software, the gray value of the map obtained by the Iba-1 (or 3-NT) positive areas in hippocampus and cortex of brain tissue slices were calculated. In ImageJ, load the image and adjust brightness. Apply thresholding to highlight Iba-1 (or 3-NT) positive areas. Then, perform particle analysis to get the area measurement, and record the data.

### 4.5. Nissl Staining

In order to observe the damage of neurons in rat brain tissue, the Nissl staining method was used to stain rat brain tissue, and the cell morphology was observed under an inverted microscope (Nikon Instruments Co., Ltd., Shanghai, China).

### 4.6. Cells

BV2 cells were obtained from the Cell Resource Center, Peking Union Medical College, and primary microglia were extracted from 1-day-old SD rats (the extraction method can be found in the Supplementary Materials) [46,47]. The cells were cultured in DMEM with 10% FBS supplemented with 1% P/S and were incubated at 37 °C with 5% CO_2_. The cells were modeled with LPS, and then cultured with rhein of different concentrations. PBS was added to dissolve LPS at a ratio of 1:1000 to prepare a solution with a concentration of 1 mg/mL, and the final treatment concentration was 1 μg/mL.

PC12 cells were obtained from the National Infrastructure of Cell Line Resource. BV2 microglia and PC12 cells were inoculated on different culture plates and cultured separately. After administration of BV2 microglia, the supernatant of BV2 microglia in each group was taken and centrifuged at 149× *g* for 5 min to remove the cells in the supernatant, and the conditioned medium was obtained. After incubating each group of conditioned media with PC12 cells for 12 h, PC12 cell plates were taken for subsequent experimental determination.

### 4.7. Cell Viability Assay

Previous reports showed that 80 μM of rhein significantly reduced the cell viability compared to control group [18]. In our study, BV2 cells or primary microglia (5 × 10^3^) were inoculated in a 96-well plate, cultured with rhein of different concentrations (0 μM, 5 μM, 10 μM, 25 μM, 50 μM, 75 μM) for 6 or 12 h, added with MTT solution, and incubated in the incubator for 4 h. Then, the supernatant was removed and 100 μL dimethyl sulfoxide (DMSO) was added. Optical density (OD) was detected at 490 nm.

### 4.8. NO and IL-1β Determination

Cells were transplanted into 24-well plates at a concentration of 1.5 × 10^5^ per well and, after LPS and rhein treatment, the supernatant of medium was collected and measured by commercial kit for NO level (Nanjing Jiancheng Bioengineering Institute, Nanjing, China). After being treated with LPS and cultured with Rhein, IL-1β was determined by mouse IL-1β enzyme-linked immunosorbent assay kit (Proteintech Group, Inc., Rosemont, IL, USA).

### 4.9. Apoptosis Detection

The apoptosis of PC12 cells was detected by Annexin V-FITC/Propidium Iodide (PI) staining. PC12 cells cultured in conditioned medium were washed twice in PBS, and then 195 μL Annexin V-FITC binding solution, 5 μL Annexin V-FITC and 10 μL PI staining solution were added in turn. The cell culture plate was wrapped with tin foil, incubated for 20 min at room temperature in the dark, and fluorescent pictures of the cells were immediately taken with a fluorescent inverted microscope (Nikon Instruments Co., Ltd., Shanghai, China).

### 4.10. Western Blotting

The BV2 microglia (2 × 10^5^) were grown to 80% confluence in complete media. After treatment with LPS and rhein, the cells were washed three times with PBS. The cell lysate was scraped from the bottom of the plate with scrapers. Then the cell lysate was transferred into a 1.5 mL centrifuge tube, and split for 30 min. The cell lysate was centrifuged at 14,000 rpm at 4 °C for 15 min. The total protein concentration was determined by BCA kit, and after leveling the protein concentration, iNOS, GLS1, GOT1, and IL-1β were detected by 10% SDS-PAGE gels, and Arg-1 was determined by 12% SDS-PAGE gels. Table 1 shows the detailed antibody information.

The extraction method of brain tissue proteins is described in the Supplementary Materials.

### 4.11. Metabolomics

#### 4.11.1. Sample Preparation

For untargeted metabolomics, primary microglia (1 × 10^6^/sample) were inoculated in a 6-well plate, cultured with complete media. After modeling with LPS, the metabolites were extracted and analyzed.

For Metabolomics, primary microglia (5 × 10^5^/sample) were inoculated in a 12-well plate, cultured with complete media. For metabolic flux analysis, BV2 cells (4 × 10^5^/sample) were cultured in medium containing [U-^13^C_5_]-glutamine and ^15^N-glutamine (4 mM). After modeled with LPS, microglial were cultured with 50 μM Rhein, and then the metabolites were extracted and analyzed.

Then cells were washed 3 times with PBS and quenched with 80% methanol. Cells were scraped off with scrapers and transferred to a 1.5 mL centrifuge tube, for liquid nitrogen freeze-thaw cycles 3 times. The supernatant was dried under nitrogen gas, and then equalized by 80% methanol. The supernatant was used for the determination of untargeted metabolomics, targeted metabolomics, and metabolic flux experiments.

#### 4.11.2. Determination Condition

The method of derivatization before determination of organic acids is described in the Supplementary Materials.

For untargeted metabolomics, the UPLC-Q-TOF/MS analysis was performed on Dionex UltiMate 3000 liquid phase system equipped with Q Exactive high-resolution mass spectrometer (Thermo Fisher Scientific, Waltham, MA, USA). Chromatographic separation was performed at 40 °C on ACQUITY UPLC BEH Amide column (2.1 × 100 mm, 1.7 μm, Waters, Bethesda, MD, USA). The mobile phase is composed of 0.1% ammonium formate-95% acetonitrile (A) and 0.1% ammonium formate-water (B). The elution program was as follows: 0–1 min, 95% A; 1–7 min, 95–50% A; 7–9 min, 50% A; 9–9.1 min, 50–95% A; 9.1–12 min, 95% A. The flow rate was set to 0.3 mL·min^−1^ and the injection volume was 5 μL. The mass range was 50–1500 *m*/*z*.

For targeted metabolomics, organic acids were determined on ACQUITY UPLC BEH C8 column (2.1 × 100 mm, 1.7 μm, Waters, UK) at 45 °C. The mobile phase was composed of 0.1% formic acid–water (A) and methanol–isopropanol (4:1) (B). The elution program was as follows: 0–2 min, 95–85% A; 2–9 min, 85–45% A; 9–10 min, 45–0% A; 10–11 min, 0% A; 11–11.1 min, 0–95% A; 11.1–13 min, 95%A. The flow rate was set to 0.3 mL·min^−1^ and the injection volume was 5 μL. An electrospray ionization source (ESI) was used in the negative mode. Amino acids were determined on ACQUITYUPLC HSS T3 column (2.1 × 100 mm, 1.7 μm, Waters, UK) at 50 °C. The mobile phase is composed of 0.1% formic acid-water (A) and acetonitrile (B). The elution program was as follows: 0–2 min, 95–85% A; 2–9 min, 85–45% A; 9–10 min, 45–0% A; 10–11 min, 0% A; 11–11.1 min, 0–95% A; 11.1–13 min, 95% A.

For metabolic flux experiments, the UPLC-Q-TOF/MS analysis was performed on a Thermo Scientific liquid phase system equipped with a Q Exactive high-resolution mass spectrometer. Organic acids were determined on an ACQUITY UPLC BEH C18 column (2.1 × 100 mm, 1.7 μm, Waters, UK) at 38 °C. The mobile phase composed of 0.1% formic acid–water (A) and methanol–isopropanol (4:1) (B). The elution program was as follows: 0–12 min, 95–0% A;12–13 min,0% A; 13–13.1 min, 0–95% A; 13–14 min, 95% A. The flow rate was set to 0.2 mL·min^−1^ and the injection volume was 8 μL. ESI was used in negative mode. Amino acids were determined on an ACQYITY UHPLC BEH amide column (2.1 × 100 mm, 1.7 μm, Waters, UK) at 38 °C. The mobile phase was composed of 0.1% formic acid–water (A) and 0.1% formic acid–acetonitrile (B). The elution program was as follows: 0–7 min, 80–55% A; 7–8 min, 55% A; 8–8.1 min, 55–80% A; 8.1–10 min, 80%. ESI was used in positive mode.

The Normalized Collision Energy (NCE) of the metabolites in metabolic flux experiments is provided in the Supplementary Materials.

### 4.12. Data Analysis and Statistical Analysis

GraphPad Prism 6.02 software was used for statistical analysis. All data are presented as mean ± SEM. Differences between multiple groups were examined using one-way ANOVA. The LSD-*t*-test was adopted to compare the data between the two groups. The significance levels were * *p* < 0.05. Metabolomics data analysis was conducted in the Progenesis QI software (version 3.0.3) and SIMCA-P (version 13.0). The heatmap and pathway analysis of differential metabolites were generated on MetaboAnalyst 6.0 (https://www.metaboanalyst.ca/, accessed on 3 November 2024).

## 5. Conclusions

In this study, we characterized a mechanism that could explain the anti-neuroinflammatory effect of rhein. We showed that rhein inhibits NO production by regulating the glutamine–aspartate–arginine metabolic pathway in activated microglia, thereby inhibiting neuroinflammation and improving neuronal damage in AD. This study shows for the first time that rhein plausibly exerts anti-neuroinflammatory activity through modulation of the glutamine–aspartate–arginine–NO metabolic pathway. These findings provide new possibilities for the pharmacological effects of rhein in the treatment of AD.

## Figures and Tables

**Table 1:** Detailed antibody information.

Antibody	Source	Dilute	Company
iNOS	Rabbit	1/2000	(Proteintech Group, Inc., Rosemont, IL, USA)
Arg-1	Rabbit	1/10,000	
GLS1	Rabbit	1/5000	
GOT	Rabbit	1/2000	
IL-1β	Rabbit	1/1000	
Beta-Actin Antibody	Mouse	1/10,000	
HRP-anti-rabbit IgG	Goat	1/2000	
HRP-anti-mouse IgG	Goat	1/2000	

## Data Availability

The original contributions presented in the study are included in the article/Supplementary Materials. Further inquiries can be directed to the corresponding authors.

## Supplementary Materials

The supporting information can be downloaded at: https://www.mdpi.com/article/10.3390/ijms26136404/s1.

## Abbreviations

AD, Alzheimer’s disease; NO, nitric oxide; TLR, Toll-like receptors; LPS, lipopolysaccharide; α-KG, α-ketoglutarate; HIF1α, hypoxia-inducible factor 1α; iNOS, inducible nitric oxide synthase; IL-1β, interleukin-1β; NCE, Normalized Collision Energy; ESI, electrospray ionization source; OPLS-DA, Orthogonal partial least square discriminant analysis; NPA, 3-nitropropionic acid; AOAA, amino-oxy-acetic acid hemi-hydrochloride.
