# Supplementary material for: Rhein Inhibits Microglia-Mediated Neuroinflammation and Neuronal Damage of Alzheimer’s Disease via Regulating the Glutamine–Aspartate–Arginine–NO Metabolic Pathway
Source: Int J Mol Sci. 2025 Jul 3;26(13):6404. doi: 10.3390/ijms26136404 (PMC12249630; doi:10.3390/ijms26136404)
Supplement: Supplementary file 1 [file ijms-26-06404-s001.zip › ijms-3669381-supplementary.pdf]

Supplementary materials

Materials and Methods

Extraction of primary microglia

Primary microglia cultures were prepared from hippocampal tissues of 1-day-old neonatal SD rats. The cerebral cortex, devoid of meninges and blood vessels, were digested with 0.125% Trypsin-EDTA for 15 minutes at 37°C. After centrifugation (1000 rpm, 15 minutes), cells were collected and re-suspended in DMEM with 20% FBS. Then the cell suspension was passed through a 70 μm mesh cell strainer and plated in T25 culture flask with vented caps to culture.

Metabolomics

Derivatization of organic acid and amino acid

For the detection of organic acids in targeted metabonomic, the organic acids were derivatized by the following steps. Briefly, 50 μL of cell samples or a standard solution, 100 μL of 50 mM 3-NPH in 75% methanol, 50 μL of 30 mM EDC in 75% methanol, and 50 μL of 7.5% pyridine in 75% methanol were mixed and allowed to react, either in a thermomixer at 30 °C for 30 minutesfor quantitation of α-ketoglutarate, succinate, fumarate, malate, and oxaloacetate. After this reaction, 50 μL of 2 mg/mL BHT in methanol was instantly added to these solutions, which were then diluted with 750 μL of water. Then vortex for 10s, centrifugation at 13000 rpm for 10 minutes, the supernatant was used to detect [1].

For the detection of amino acids in targeted metabonomic, 20 μL cell sample

solution, 5 μL 10 μg/mL internal standard, 40 μL isopropanol (1% formic acid) were mixed. Taken 10 μL upper solution, added AccQ-TagUltra derivatising reagent and derivatise at 50°C for 10 minutes. After this reaction, it was diluted 10 times with ultra-pure water to be detected [2].

For the detection of organic acids in metabolic flux analysis, 50 μL cell sample solution, 50 μL 50 mM 3-NPH, 50 μL 30 mM EDC, and 50 μL 1.5% pyridine were mixed and allowed to react, either in a thermomixer at 30°C for 30 minutes for detection of α-ketoglutarate, succinate, fumarate, malate, and citric acid. After this reaction, the solution was dried with nitrogen, and then added 2 mL water. It was extracted twice with chloroform, and taken trichloromethane layer. Then the trichloromethane was dried with nitrogen, and redissolved with 750 μL of 20% methanol. The solution was centrifugated at 13000 rpm for 10 minutes, and the supernatant was taken for detection [1].

The Normalized Collision Energy (NCE) of metabolites in metabolic flux

The Normalized Collision Energy (NCE) of the metabolites in metabolic flux experiments is shown as follows.

[U-$^{13}C_5$]-glutamine labeling

**Compound**	***m/z***	**$^{13}C_U$- *m/z***	**NCE**
α-ketoglutarate	550.14403	555.16081	30
Succinate	387.10586	391.11928	27
Fumarate	385.09020	389.10363	30
Malate	403.10077	407.11419	10
Citric acid	596.14951	602.16964	10
Glutamate	148.06043	153.07721	25
Aspartate	134.04478	138.05820	25

^{15}N-Glutamine labeling

**Compound**	***m/z***	**^{15}N- *m/z***	**NCE**
Glutamate	148.06043	149.05747	25
Aspartate	134.04478	135.04182	25
Arginine	175.11895	176.11599	30

Western blotting

The right hippocampal tissue of rats in each group was weighed. Protease inhibitor PMSF and tissue lysate RIPA (1 mg/10 μL) were added to the hippocampal tissue. The tissue was homogenized using an electric homogenizer. The samples were placed at 4 °C for cleavage for 30 minutes. Then the solution was centrifuged at 14 000 rpm at 4 °C for 15 minutes, and the supernatant was placed in a 1.5 mL centrifuge tube. The total protein concentration was determined by BCA kit. iNOS, GLS1, GOT1, and IL-1β were detected by 10% SDS-PAGE gels, and Arg-1 was determined by 12% SDS-PAGE gels.

Supplementary Figure

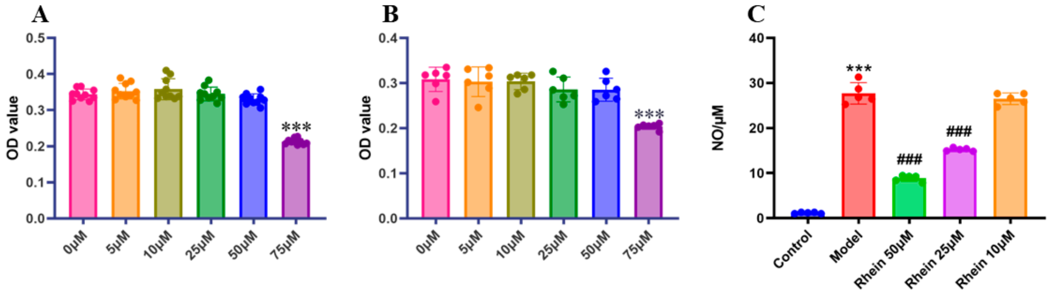

Figure S1. Rhein inhibits LPS-induced microglial activation-mediated inflammation. The cell viability assessed using MTT pretreated with or without different concentrations of rhein (0, 5, 10, 25, 50, and 75 μM) for 6h (**A**) and 12h (**B**) in primary microglia. Compared with the control group (0 μM group), $^{***}p < 0.001$. (**C**) The effect of Rhein on NO secretion of primary microglia. Compared with the control group, $^{***}p < 0.001$. Compared with the model group, $^{\#\#\#}p < 0.001$.

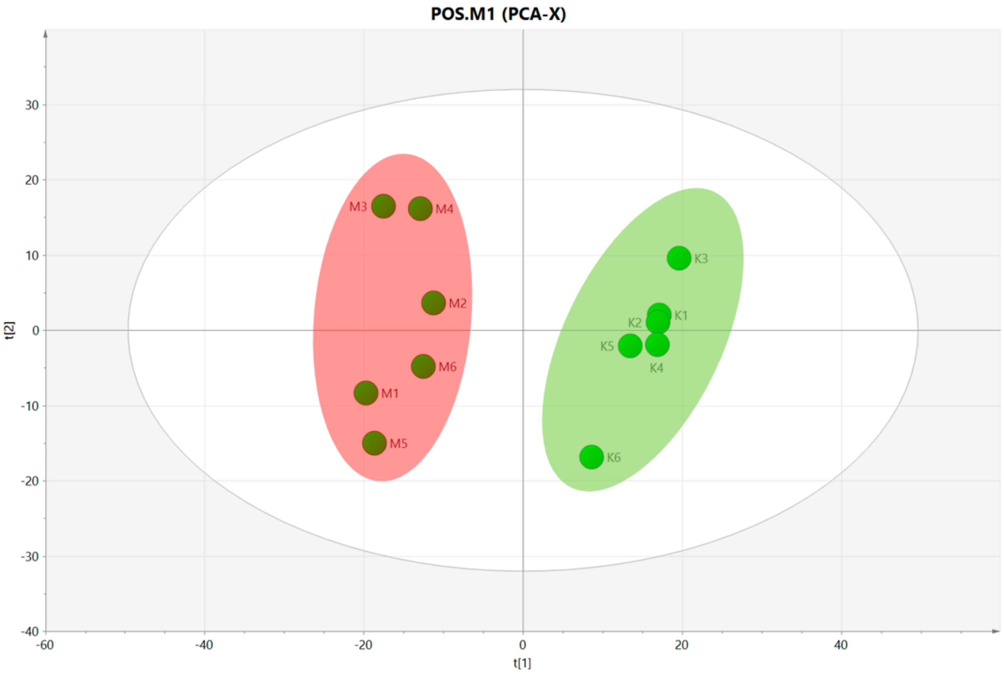

Figure S2. PCA of primary microglia between control group and LPS group.

Supplementary Table

Table S1. The differential metabolites between the control group and the model group of primary microglia.

No.	t_R	*m/z*	Name	VIP
1	6.38	258.1091	Glycerophosphocholine	23.45
2	6.62	184.0728	Phosphorylcholine	16.42
3	7.37	399.1433	S-Adenosylmethionine	12.66
4	6.55	176.1025	Citrulline	11.87
5	5.05	162.1118	Carnitine	9.94
6	6.66	188.1751	N1-Acetylspermidine	9.78
7	8.52	146.1648	Spermidine	8.64
8	4.89	132.1016	Leucine	7.56
9	7.20	147.1124	Lysine	7.37
10	4.47	146.1171	4-Trimethylammoniobutanoic acid	7.14
11	5.97	132.0764	Beta-Guanidinopropionic acid	6.52
12	2.26	522.3539	LysoPC(18:1/0:0)	6.37
13	4.95	235.1645	Norvaline	6.37
14	0.98	524.3698	LysoPC(0:0/18:0)	6.18
15	3.85	137.0454	Threonic acid	5.63
16	7.78	428.0356	dGDP	5.54
17	2.39	136.0614	Adenine	5.12
18	6.66	203.1497	Asymmetric dimethylarginine	4.45
19	6.11	120.0654	Threonine	3.97
20	5.06	150.058	Methionine	3.77
21	5.55	203.2225	Spermine	3.66
22	4.36	130.0496	Pyroglutamic acid	3.27
23	6.40	148.0600	Glutamate	3.23
24	1.36	123.0551	Niacinamide	3.20
25	5.43	116.0706	Proline	3.13
26	2.61	502.2913	LysoPE(20:4/0:0)	2.98

Table S1 (Continued)

No.	t_R	*m/z*	Name	VIP
27	2.68	480.3071	LysoPE(18:1/0:0)	2.93
28	6.69	170.0920	3-Methylhistidine	2.56
29	7.91	613.1577	Oxidized glutathione	2.34
30	7.00	89.1077	Putrescine	2.32
31	2.56	526.2914	LysoPE(22:6/0:0)	1.99
32	6.7	134.0445	Aspartate	1.96
33	6.19	147.0761	Glutamine	1.90
34	7.59	489.1135	Citicoline	1.87
35	5.46	182.0808	Tyrosine	1.84
36	2.9	452.2757	LysoPE(0:0/16:1)	1.80
37	5.11	162.0758	Aminoadipic acid	1.77
38	6.84	189.1592	N6,N6,N6-Trimethyl-L-lysine	1.73
39	6.15	189.1341	Homo-L-arginine	1.72
40	6.97	161.128	Isoputreanine	1.71
41	2.16	568.3381	LysoPC(22:6/0:0)	1.67
42	7.96	405.0083	Uridine 5'-diphosphate	1.61
43	2.18	130.086	Pipecolic acid	1.58
44	5.04	219.0969	Glutamylalanine	1.55
45	7.55	335.0727	Glycerophosphoinositol	1.53
46	7.20	191.1134	N(omega)-Hydroxyarginine	1.52

References

[1] Han J, Gagnon S, Eckle T, Borchers CH. Metabolomic analysis of key central carbon metabolism carboxylic acids as their 3-nitrophenylhydrazones by UPLC/ESI-MS. *Electrophoresis*. 2013;34(19):2891-2900.

[2] Gray N, Zia R, King A, et al. High-Speed Quantitative UPLC-MS Analysis of Multiple Amines in Human Plasma and Serum via Precolumn Derivatization with 6-Aminoquinolyl-N-

hydroxysuccinimidyl Carbamate: Application to Acetaminophen-Induced Liver Failure. *Anal Chem*. 2017;89(4):2478-2487.
